# Impact of Two-Way FDI on China’s Environmental Quality: The Perspective of Environmentally Cleaner Production and End Treatment

**DOI:** 10.3390/ijerph20054320

**Published:** 2023-02-28

**Authors:** Zhenya Zhang, Wanping Yang, Dong Li, Yajuan Wang

**Affiliations:** 1 School of Economics and Finance, Xi’an Jiaotong University, Xi’an 710064, China; 2 School of Management, Fudan University, Shanghai 200433, China

**Keywords:** inward foreign direct investment, outward foreign direct investment, environmental quality, environmentally cleaner production, environmental end treatment, Dagum Gini coefficient, system-generalised method-of-moments

## Abstract

While the rapid development of two-way foreign direct investment (FDI) has boosted China’s economic growth, its impact on environmental quality is uncertain. Based on provincial panel data from China covering the period from 2002 to 2020, this paper proposes an environmental quality assessment index system for China from two aspects: environmentally cleaner production and environmental end treatment. The comprehensive environmental quality index (EQI), environmentally cleaner production index (EPI), and environmental end treatment index (ETI) were all measured, with the geographic information system tool and Dagum Gini coefficient used to analyse the indicators’ differences using a system-generalised method-of-moments (SYS-GMM) estimation to study the impact of two-way FDI on environmental quality in various regions across China. The results demonstrate that during the sample period, inward FDI positively impacted environmental quality and cleaner production but had a negative impact on environmental end treatment. Outward FDI significantly promoted EQI, EPI, and ETI, and the interaction between inward FDI and outward FDI positively impacted environmental quality and environmentally cleaner production, while it negatively impacted environmental end treatment. This indicates that under two-way FDI, China’s relationship with environmental quality has gradually evolved from ‘pollution first and then treatment’ to ‘green development of cleaner production’.

## 1. Introduction

Promoting a high-level opening-up has been an important driving force of China’s sustained and rapid high-quality development. The theme of the G20 Rome Summit in 2021 was ‘People, the Earth, and Prosperity’, which placed climate change and environmental protection at the top of the agenda. In November 2022, the theme of the G20 Bali Summit in Indonesia was ‘Common Recovery, Strong Recovery’, which continued to emphasise the goal of strengthening the degree of attention paid to multilateral trade and environmental protection. The report of the 20th National Congress of the Communist Party of China (CPC) in October 2022 listed promoting a high-level opening-up and accelerating China’s transformation into a powerful trading nation as a key element of the construction of a modern economic system. It also cited accelerating the green transformation of China’s development mode and the prevention and control of environmental pollution as key aspects of China’s path to modernisation.

In recent years, China has adhered to a strategy of ‘bringing in’ and ‘going out’ and has gradually formed an open pattern of two-way investment and mutual assistance. In 2020, China’s influx of foreign capital reached USD 163 billion, allowing China to surpass the United States as the world’s premier foreign capital destination. In 2021, the utilisation of China’s inward foreign direct investment (IFDI) rose to USD 173.48 billion from USD 52.743 billion in 2002, an increase of 228.9%. China’s outward foreign direct investment (OFDI) flow reached USD 178.82 billion in 2021, emphasising China’s rapid growth in OFDI of 24.69% annually for 20 consecutive years, from 2002 to 2021. However, the negative impact of increased investment on environmental quality cannot be ignored. Excessive energy consumption is a major issue [1]; air, water, and soil pollution are also serious problems [2,3,4], as China now ranks first in the world for carbon emissions [5]. However, the impact of two-way FDI on environmental quality currently remains unclear. On one hand, the rapid increase in two-way FDI in China has promoted the rapid expansion of the industrial scale, which has generated a marked increase in both natural resource consumption and environmental pollution, presenting an unprecedented challenge to the ecological environment. On the other, the promotion of two-way FDI has also led to the transfer of superior technology and management practices, which can improve pollution control and generate cleaner production, leading to an improvement in environmental quality. In light of these conflicting developments, this study addresses the impact of two-way FDI on China’s environmental quality, explores the factors affecting the improvement of environmental quality in various regions, and, through empirical analysis, reveals whether China’s development model has changed from an expansive development model focused on ‘pollution before treatment’ to a strong, sustainable development model centred around ‘clean production’. This paper is expected to have a certain significance for China to realise ‘promote green development and achieve high-level opening-up’ goals.

## 2. Literature Review

Bidirectional FDI refers to the combination of both IFDI and OFDI. With regard to the impact of foreign direct investment (FDI) on the environmental quality of host countries, the most famous hypotheses are ‘pollution paradise’ and ‘pollution halo’.

Many researchers have argued that developed countries have actively transferred their domestic industries with high pollution and high emissions to other underdeveloped regions, thus empirically proving the ‘pollution paradise’ hypothesis [6]. Some researchers have found that developing countries and regions voluntarily lower environmental standards and environmental regulation thresholds to obtain foreign capital and, in doing so, address their own capital shortcomings [7]. This is the ‘race to the bottom’ phenomenon in developing countries, which creates a long-term balance between IFDI and pollution [8]. Ali et al. found that IFDI significantly increased the ecological footprint of Islamic countries, thus hindering the improvement of environmental quality [9]. Based on the data of N-11 countries from 1980 to 2018, Aslan et al. adopted a panel vector autoregression model and found that IFDI has led to increased pollution in these countries [10]. It was also found that the overall improvement in the economic level was less than the investment in environmental governance. Since then, many relevant researchers have supported the ‘pollution paradise’ hypothesis, believing that the inflow of foreign capital will reduce the local environmental quality [11,12].

The ‘pollution halo’ hypothesis states that IFDI can improve the environment of host countries through technology spillovers, demonstrations of production technology or management experience, and stricter environmental standards. Some researchers have found that the level of technology and mature management experience of transnational enterprises can play an exemplary role for enterprises in the host country, which can, in turn, create an improvement in environmental quality [13,14]. Sitthivanh and Srithilat studied the impact of IFDI on the environmental quality of the Association of Southeast Asian Nations (ASEAN) countries. The results demonstrate that introducing foreign capital improved local cleaner production technology and helped improve the region’s overall environmental quality [15]. Through an empirical analysis, Ayamba et al. found that IFDI is the most important method of technology transfer for investing countries, one which can effectively upgrade the technology of investment inflows and effectively improve local pollution problems [16]. A number of studies have constructed China’s green economy index and concluded that foreign direct investment promotes green economic growth through independent innovation and inhibits green economic growth through imitation innovation [17].

The existing research on OFDI has mainly focused on its impact on economic growth, its effect on the home country’s production efficiency, developments of cleaner production technology, and other positive effects, while less focus has been placed on the environmental impact on the home country. Some researchers have proven that OFDI positively affects domestic production and operations by improving enterprise productivity and the business scale [18]. The reverse technology spillover effect of OFDI also has a significant positive effect on domestic innovation capacity [19], but this effect is limited by the absorptive capacity of the home country’s enterprises, OFDI, and differences in market competition intensity [20]. Most studies confirm that OFDI effectively allows enterprises to overcome internal resource constraints and leapfrog the technological frontier [21]. Some studies have found that OFDI has had a significant positive impact on the increase in China’s green total factor productivity and has also promoted the impact of environmental regulations on China’s green total factor productivity through intermediary effects [22].

However, some researchers have arrived at the opposite conclusion. For example, Hao concluded that China’s OFDI has increased domestic environmental pollution by increasing its economic scale [23]. The empirical results demonstrate that investment in environmental control cannot reduce but, rather, increases China’s carbon emissions. Some researchers have proposed that both OFDI flow and stock have significantly increased SO_2_ emissions in China, mainly because OFDI has not improved China’s emission reduction quality through reverse technology spillover [24]. Meanwhile, some researchers remain uncertain as to the impact of OFDI on the environment. For example, through empirical research on the impact of OFDI on China’s domestic carbon dioxide emissions, Ouyang et al. found that an increase in OFDI can significantly improve the air pollution level of local cities and reduce pollution spillover from local cities to surrounding cities [25].

Several researchers have studied the environmental effects of two-way FDI. Mu-hammad and Khan argued that bilateral FDI could promote economic growth in Asian countries and improve the local ecological environment through the use of clean energy and clean production technology [26]. Gong and You used data from 30 provinces and cities in China from 2004 to 2017 and concluded that two-way FDI inhibits the progress of regional environmental technologies [27]. Some researchers believe that OFDI can significantly improve the ecological environment, whereas IFDI is detrimental to environmental improvement. Other researchers have found that China is now in the early stage of OFDI development, and the emission reduction effect of OFDI is not as obvious as that of IFDI [28]. At the same time, other studies have found that both IFDI and OFDI have significantly promoted the efficiency of green innovation in China’s manufacturing industry and also promoted the improvement of regional environmental quality through interaction with environmental regulations [29].

In the above-mentioned studies, it can be observed that there are two kinds of controversies surrounding the impact of IFDI on the environmental quality of the host country over a lengthy time period: ‘pollution paradise’ and ‘pollution halo’, on which no definite conclusion has yet been reached. There have been fewer relevant studies carried out on the environmental effects of OFDI, and there is no consensus on whether the impact of OFDI on the environment of the host country is positive or negative. There is also no consensus on whether the impact of OFDI on the environment of the home country is positive or negative. Existing studies are less concerned with the mechanism that affects the impact of two-way FDI on environmental quality, while for emerging market economies, both FDI and OFDI play an important role in promoting economic growth and integrating these economies into the global market. Studying the impact of either party on environmental quality in isolation is thus one-sided and cannot provide useful references and lessons for real economic activities.

Existing studies study the environmental effects of FDI from the perspective of pollution emissions, ignoring the process of pollution production and its relationship to pollution control and, in turn, to pollution emission, which makes it difficult to identify in which part of the production process the environmental effects of two-way FDI actually appear. Therefore, it is necessary to analyse the environmental effects of two-way FDI from two perspectives: pollution production and pollution control, and to investigate whether two-way FDI fundamentally achieves clean production or pollution first and then control. China is a nation that is characterised by large regional differences. The industrial structure, technological innovation, and resource endowment in the eastern region differ greatly from those available in the central and western regions, which may lead to regional heterogeneity in the environmental effects of two-way FDI. Thus, it is necessary to study the environmental effects of two-way FDI in different regions.

## 3. Materials and Methods

### 3.1. Study Motivation

**Figure 1:**
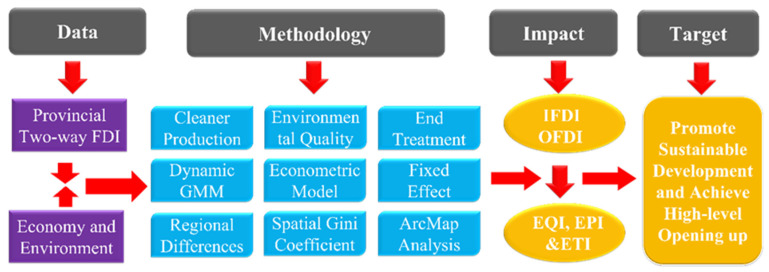
Research framework.

While reviewing the existing literature, it was noted that while the relevant research results on the impact of IFDI and OFDI on environmental quality were undoubtedly valuable, a comprehensive evaluation of China’s environmental quality, based on the concept of strong sustainability, had not been fully realised and the evaluation index system was not yet sufficiently comprehensive. Research on the evolution of environmental quality, utilising the concept of time and space, still needs to be improved. Therefore, this study expands upon the existing literature in the following ways: First, based on the concept of strong sustainability, a systematic and scientific comprehensive evaluation system of environmental quality is built through the two dimensions of an ‘environmentally clean production index’ and an ‘environmental end treatment index’. Second, the ‘vertical and horizontal’ dispersion method is used to evaluate China’s environmental clean production index (EPI), environmental end treatment index (ETI) and comprehensive environmental quality index (EQI). Third, the temporal evolution and spatial differences between the EQI, EPI and ETI in China’s provinces are analysed using ArcGIS and Dagum Gini coefficients to interpret and, in turn, describe the evolution trend. Fourth, using the dynamic-generalised method-of-moments (GMM) model, the impact of China’s IFDI and OFDI on environmental quality is revealed in a panoramic view, and the factors affecting the improvement of environmental quality are explored. The research framework is illustrated in Figure 1.

The possible contribution of our paper to existing theory is mainly twofold. First, it compares relevant studies to explore and improve the effectiveness and accuracy of the comprehensive assessment of environmental quality [30]. According to the specific requirements of the report of the 20th National Congress of the CPC, such as ‘adhering to the integrated protection and systematic governance of mountains, rivers, forests, fields, lakes, grass and sand’ and ‘promoting carbon reduction, pollution reduction, green expansion and growth in a coordinated manner, and promoting ecological priority, conservation and intensive, green and low-carbon development’, this study focuses on the two dimensions of an ‘environmentally cleaner production index’ and an ‘environmental end treatment index’ and constructs an evaluation index system that reflects the internal requirements for the construction of an ecological civilisation.

Second, in terms of research methodology, this paper uses the ‘vertical and horizontal’ dispersion method to calculate the level of ‘emission reduction’ and ‘end treatment’ of pollution in each region and describes its space-time evolution process. Using the dynamic GMM model, this study analyses the environmental impact of China’s two-way FDI from the perspective of pollution production and pollution control and discusses whether China’s two-way FDI has promoted the improvement of environmental quality. This process aims to identify whether China’s development model focuses extensively on ‘pollution before treatment’ or instead represents the development of a strong, sustainable emission reduction strategy focused on the pollution source directly.

### 3.2. Method

#### 3.2.1. Comprehensive Evaluation Method

Taking 2002 as the base period, we first standardised the original data through the fixed base efficiency coefficient method and then determined the weight of the basic indicators by using vertical and horizontal grading methods [31]. Finally, each province’s comprehensive EQI, EPI and ETI were determined using the linear weighting method. We used Equation (1) to process the original data:

(1) $$s_{ij}\left(t_{k}\right)=\left\{\begin{array}{c} \frac{\max\left[x_{j}\left(t_{0}\right)\right]-x_{ij}\left(t_{k}\right)}{\max\left[x_{j}\left(t_{0}\right)\right]-\min\left[x_{j}\left(t_{0}\right)\right]},\;x_{j}\;is\;a\;negative\;indicator\\ \frac{x_{ij}\left(t_{k}\right)-\min\left[x_{j}\left(t_{0}\right)\right]}{\max\left[x_{j}\left(t_{0}\right)\right]-\min\left[x_{j}\left(t_{0}\right)\right]},\;x_{j}\;is\;a\;positive\;indicator \end{array}\right.$$

where *x_ij_*(*t_k_*) and *s_ij_*(*t_k_*) represent the original and standardised values of the *j*th index of the *i*th province in the *t_k_* year, respectively, and max[*x_j_*(*t*_0_)] and min[*x_j_*(*t*_0_)] represent the maximum and minimum values of the *j*th index of all provinces in the base period, respectively.

Second, we estimated the objective weight coefficient *ω*. If the set of objects to be evaluated is *s* = { *s*_1_, *s*_2_, … *s*_n_ }, the evaluation function of year *t_k_* is

(2) $$y_{i}\left(t_{k}\right)=\sum_{j=1}^{m}\omega_{j}X_{ij}\left(t_{k}\right),\;k=1,2,\ldots N,\;i=1,2\ldots n$$

where $\omega ={\left(\omega_{1},\omega_{2},\ldots \omega_{m}\right)}^{T}$ is the weight coefficient vector. The specific formula to describe the difference between the evaluation objects by the sum of the squares of the total deviations of *y_i_*(*t_k_*) is

(3) $$\left\{\begin{array}{c} \delta^{2}=\sum_{k=1}^{N}\sum_{i=1}^{n}[{\left(y_{i}\left(t_{k}\right)-\bar{y}\right]}^{2}=\sum_{k=1}^{N}\sum_{i=1}^{n}[{\left(y_{i}\left(t_{k}\right)\right]}^{2}=\sum_{k=1}^{N}\left[\omega^{T}H_{k}\omega \right]=\omega^{T}\sum_{k=1}^{N}H_{k}\omega =\omega^{T}H\omega \\ H=\sum_{k=1}^{N}H_{k};\;H_{k}=A_{K}^{T}A_{k}\\ A_{k}=\left\{\left(\begin{array}{ccc} x_{11}\left(t_{k}\right) & \cdots & x_{1m}\left(t_{k}\right)\\ \vdots & \ddots & \vdots \\ x_{n1}\left(t_{k}\right) & \cdots & x_{nm}\left(t_{k}\right) \end{array}\right)\right\},\;k=1,2,\ldots N \end{array}\right.$$

where *A*_k_ is the matrix form of Equation (2), that is, *Y* = *Aω*. *H_k_* is the *m* × *n*-order symmetric matrix, *k* = 1, 2, … *N*. *H* is the m × m-order symmetric matrix. *λ_max_*(*H*) is the standard eigenvector corresponding to the maximum eigenvalue of the real symmetric matrix *H*. *ω* is the combined weight vector determined by normalising the standard feature vector *λ_max_*(*H*).

The following three conclusions can be drawn. First, if limited, *ω^T^ω* = 1 when *ω* is taken as the maximum eigenvalue for matrix *H*. When *λ_max_*(*H*) corresponds to the characteristic vector, *δ^2^* takes the maximum and has *ω^T^Hω* = *λ_max_*(*H*). Second, when *H_k_* > 0, *t_k_* (*k* = 1, 2, … *N*), at the time *t_k_*, the order of *S_i_* of the evaluated object obtained by applying the transverse scattered grade method is the same.

Third, the comprehensive assessment index of environmental quality was calculated as follows:

(4) $$Q_{i}\left(t_{k}\right)=\sum_{j=1}^{m}\omega_{j}s_{ij}\left(t_{k}\right)$$

where *Q_i_*(*t_k_*) is the assessment index of environmental quality in the *t_k_* year of the *i*th province (Table 1).

#### 3.2.2. Dagum Gini Coefficient

The Dagum Gini coefficient method [32] was used to dynamically interpret the spatial differences in China’s comprehensive environmental quality in the east, middle and west and explore the underlying reasons. According to the decomposition method proposed by Dagum, the overall Gini coefficient (*G*) can be divided into intra-regional differences (*G_w_*), inter-regional differences (*G_nb_*) and hypervariable densities (*G_t_*). This satisfies the following requirements.

(5) $$G=G_{w}+G_{nb}+G_{t}$$

Generally, the smaller the Gini coefficient, the smaller the regional difference and the stronger the regional convergence. Conversely, the larger the Gini coefficient, the weaker the regional synergy. According to Dagum’s definition and the research content of this study, the overall Gini coefficient is defined as

(6) $$G=\frac{\sum_{j=1}^{k}\sum_{h=1}^{k}\sum_{i=1}^{n_{j}}\sum_{r=1}^{n_{h}}\left|y_{ji}-y_{hr}\right|}{2n^{2}\mu }$$

For this analysis, China was divided into three major regions—the east, middle and west; *k* represents the number of regions divided, *n* represents the number of all provinces, *y_it_*(*y_hr_*) represents the comprehensive environmental quality of province *j*(*r*) in *j*(*h*), and *n_j_*(*n_h_*) represents the number of provinces in *j*(*h*). *μ_j_*(*μ_m_*) is the mean value of the EQI in region *j*(*h*). Among them, the Gini coefficient *G_jj_* of region *j* and intra-region difference *G_w_* can be expressed as Equations (7) and (9). The Gini coefficient *G_jh_* between regions *j* and *h* and the net value difference *G_nb_* between regions are given by Equations (8) and (10). The super-variable density *G_t_* can be expressed as illustrated in Equation (11).

(7) $$G_{jj}=\frac{\sum_{i=1}^{n_{j}}\sum_{r=1}^{n_{j}}\left|y_{ji}-y_{hr}\right|}{2n_{j}^{2}\mu_{j}}$$

(8) $$G_{jh}=\frac{\sum_{i=1}^{n_{j}}\sum_{r=1}^{n_{jh}}\left|y_{ji}-y_{hr}\right|}{n_{j}n_{h}\left(\mu_{j}+\mu_{jm}\right)}$$

(9) $$G_{w}=\overset{k}{\underset{j=1}{\sum }}G_{jj}p_{j}s_{j}$$

(10) $$G_{nb}=\overset{k}{\underset{j=2}{\sum }}\overset{j-1}{\underset{h=1}{\sum }}G_{jh}(p_{j}s_{h}+p_{h}s_{j})D_{jh}$$

(11) $$G_{t}=\overset{k}{\underset{j=2}{\sum }}\overset{j-1}{\underset{h=1}{\sum }}G_{jh}(p_{j}s_{h}+p_{h}s_{j})(1-D_{jh})$$

In Equation (10),

(12) $$p_{j}=n_{j}/n,\;s_{j}=n_{j}\mu_{j}/n\mu$$

In Equation (11), *D_jh_* measures the interactive impact of the comprehensive environmental quality between regions *j* and *h*. *d_jh_* refers to the difference in the comprehensive environmental quality between regions, representing the mathematical expectation of *y_it_* − *y_hr_* > 0 between regions *j* and *h*, and *P_jh_* is the super-variable first moment, representing the mathematical expectation of *y_it_* − *y_hr_* < 0 between regions *j* and *h*.

(13) $$D_{jh}=\frac{d_{jh}-p_{jh}}{d_{jh}+p_{jh}}$$

(14) $$d_{jh}=\int_{0}^{\infty }dF_{j}\left(y\right)\int_{0}^{y}\left(y-x\right)dF_{h}\left(x\right)$$

(15) $$p_{jh}=\int_{0}^{\infty }dF_{h}\left(y\right)\int_{0}^{y}\left(y-x\right)dF_{j}\left(x\right)$$

#### 3.2.3. Dynamic Panel Model

For this analysis, China was divided into east, middle and west, according to geographical location. The funds ‘introduced’ and ‘going out’ from each region were collated, while the ETI was evaluated from the EQI and the EPI to explore the environmental effects of two-way FDI and its impact on cleaner production and terminal governance capacity:

(16) $$EQI_{it}=\alpha_{0}+\beta_{1}IFDI_{it}+\beta_{2}OFDI_{it}+\mu_{i}+\xi_{it}$$

(17) $$EPI_{it}=\alpha_{0}+\beta_{1}IFDI_{it}+\beta_{2}OFDI_{it}+\mu_{i}+\xi_{it}$$

(18) $$ETI_{it}=\alpha_{0}+\beta_{1}IFDI_{it}+\beta_{2}OFDI_{it}+\mu_{i}+\xi_{it}$$

As the impact of IFDI and OFDI on the environment is often characterised by persistence, that is, changes in a given period may have an impact on the next, this study considers the lag of the explained variable as an explanatory variable to be included in the model. As the impact of IFDI and OFDI on the environment is, to some extent, interactive, the interaction term between them is added. According to the pollution haven hypothesis, the environmental Kuznets curve hypothesis and a number of previous studies, economic development level (ED), industrial structure (IND), energy structure (ES), environmental regulation (ER), research and development investment (RD), and population density (POP) also have a significant impact on the environment. Thus, these variables were selected as control variables. To reduce heteroscedasticity, variables other than percentages were logarithmically treated in the empirical testing process. Subsequently, the dynamic GMM model was set as follows:

(19) $$lnY_{it}=\alpha_{0}+\beta_{1}lnY_{it-1}+\beta_{2}lnIFDI_{it}+\beta_{3}lnOFDI_{it}+\beta_{4}lnIFDI_{it}lnOFDI_{it}+\sigma_{1}lnM_{it}+\mu_{i}+\xi_{it}$$

where subscripts *i* and *t* represent province and year, respectively, and *β* and *σ* are the spatial interaction term coefficients of the corresponding variable. *Y_it_* is the explained variable, which represents EQI, EPI and ETI. IFDI and OFDI are the core explanatory variables. *Y*_*it*−1_ represents the first-order lag term of the EQI, EPI and ETI for each province and serves as the core explanatory variable of the dynamic GMM. *M_it_* refers to the relevant control variables used to control for other exogenous factors that affect environmental quality, cleaner production and end-of-pipe treatment. *ε_it_* is the random error term. *μ_it_* refers to individual fixed effects.

### 3.3. Research Data

#### 3.3.1. Dependent Variables

EQI, EPI and ETI are the explained variables representing the environmental comprehensive quality index, EPI and ETI, respectively.

#### 3.3.2. Independent Variables

IFDI and OFDI are independent variables. Based on the ‘pollution paradise hypothesis’ and ‘pollution halo hypothesis’, they may either promote the improvement of environmental quality or lead to the deterioration of the ecological environment. The interaction terms of IFDI and OFDI were used to analyse the regulatory effect between them.

#### 3.3.3. Control Variables

The analysis controlled for several variables. Economic development level (ED), according to the environmental Kuznets inverted ‘U’ curve hypothesis, has an important impact on environmental quality [33]. For industrial structure (IND), if the development of the tertiary industry added value is at its peak and the pollution-added value is smaller than that of industry, it can promote improved environmental quality [34]. The larger the proportion of coal consumption in the energy structure (ES), the worse the environmental quality and the more serious the environmental pollution. Environmental regulation (ER) reflects the strength of local government restrictions on polluting industries and environmental management. A higher level of R&D investment (RD) can improve cleaner production and pollution control technology, which can improve environmental quality [35]. The greater the population density (POP), the fewer per capita resources in the region and, thus, the weaker the environmental carrying capacity and the easier pollution accumulates; thus, a higher POP may negatively impact EQI [36].

Table 2 lists the calculation methods and data sources for the regression variables.

### 3.4. Data Description

With reference to the criteria of the National Development and Reform Commission, we divided China into three economic regions: eastern, central and western. The eastern region includes 11 provinces and municipalities directly under the Chinese Central Government: Beijing, Tianjin, Hebei, Liaoning, Shanghai, Jiangsu, Zhejiang, Fujian, Shandong, Guangdong and Hainan. The central region comprises eight provinces: Shanxi, Jilin, Heilongjiang, Anhui, Jiangxi, Henan, Hubei and Hunan. The western region includes 11 provinces, autonomous regions, and municipalities directly under the Central Government: Inner Mongolia, Guangxi, Sichuan, Chongqing, Guizhou, Yunnan, Shanxi, Gansu, Qinghai, Ningxia and Xinjiang. Tibet, Hong Kong, Macao and Taiwan were not included in this study because their data were unavailable. We selected panel data from 30 provinces in China, covering the period from 2002–2020. The data for each indicator were collected from the China Statistical Yearbook (2003–2021) and Provincial Statistical Yearbook (2003–2021). Some missing data were supplemented by the Wind database, yearbooks, and statistical bulletins of various provinces. Table 3 presents the descriptive statistics for each variable.

## 4. Feature Fact Description and Evolution Analysis

### 4.1. Feature Fact Description

#### 4.1.1. Scale Development of IFDI

**Figure 2:**
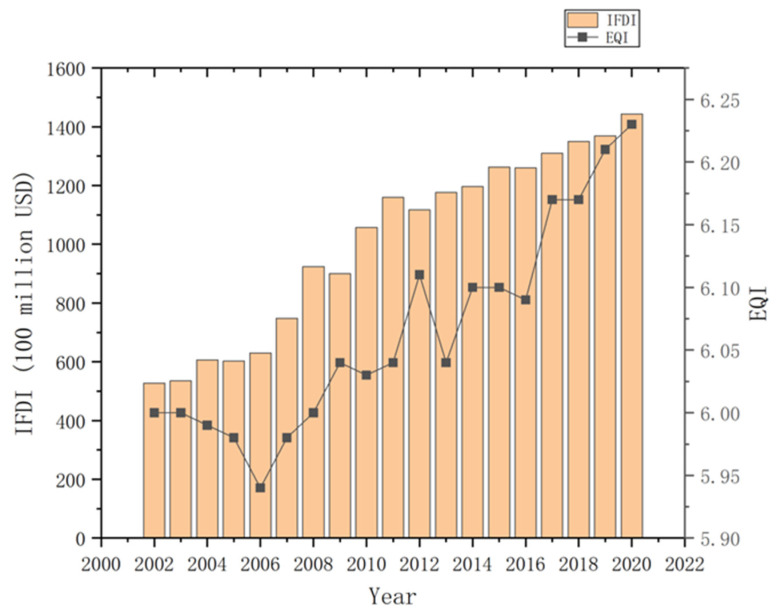
The development trend of China’s inward foreign direct investment flow in the observation period (2002–2020).

Due to its rapid, stable economic growth and loose foreign investment environment, China has continually attracted large volumes of foreign investment, gradually becoming one of the largest centres of foreign investment inflows worldwide. Figure 2 describes the developmental trend of China’s IFDI flow during the observation period (2002–2020). China’s IFDI has maintained steady growth throughout this period, demonstrating only a slight decline after 2015 and continuing to rise once more after 2016. According to data from World Investment Report 2020, released by the United Nations, the impact of COVID-19 [37] caused global IFDI to fall by 42% year-on-year in 2020. China’s use of foreign investment for the year 2020 was USD 144.369 billion, an increase of 4.51% year-on-year. China’s FDI scale ranked first in the world due to the optimisation of China’s business environment and the gradual improvement of foreign investment services.

#### 4.1.2. Scale Development of OFDI

**Figure 3:**
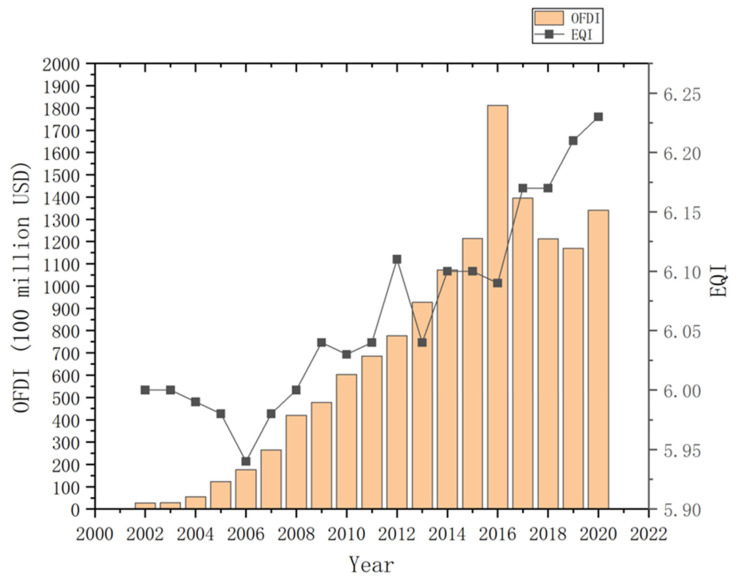
The development trend of China’s outward foreign direct investment flow in the observation period (2002–2020).

Although China’s OFDI development started late and faced many difficulties, including a lack of experience, China’s ‘going out’ strategy has since allowed its OFDI to develop rapidly. Figure 3 illustrates the development of China’s OFDI during the observation period, demonstrating the scale at which China’s OFDI flows have increased annually. China’s OFDI flow in 2020 was 56.9 times that of 2002, accounting for more than 10% of global FDI flow for four consecutive years. China’s OFDI peaked in 2016 at 181.23 billion dollars, after which it declined, with China’s OFDI also entering a stable development period. In 2020, China’s OFDI flow reached USD 153.71 billion, ranking first globally and demonstrating China’s growing influence on global OFDI and the world economy at large.

It can be seen from Figure 2 and Figure 3, with the growth of IFDI and OFDI, the change in EQI showed a decline first and then an increase. This reflects China’s transformation from a weak sustainable development concept centred on ‘economic growth’ to a strong, sustainable development concept centred on ‘ecological environment protection’.

#### 4.1.3. IFDI Regional Differences

**Figure 4:**
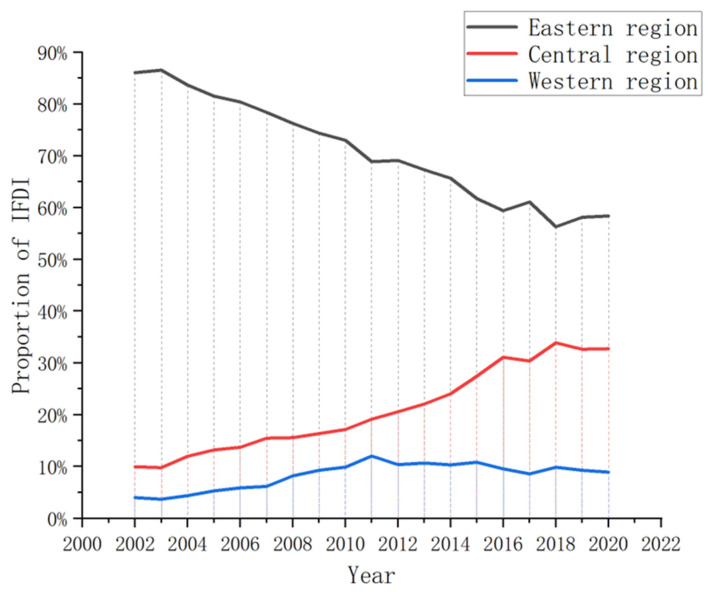
Proportion of IFDI in eastern, central and western China from 2002 to 2020.

The amount of foreign capital used in China’s eastern region is far higher than in the central and western regions. While usage is also higher in the central region than in the western region, the difference is not obvious. Figure 4 illustrates the changing trend in the proportion of foreign capital utilised in each of the three regions during the observation period. During this time, the proportion of IFDI flow accounted for by the eastern region decreased from 86.04% in 2002 to 58.36% in 2020, while for the eastern and western regions, it increased from 9.96% to 32.72% and 4.0% to 8.92%, respectively. Overall, while the actual level of foreign capital utilisation among these regions remains imbalanced, the gap is narrowing. The rapid development of the eastern region has meant that its labour costs and environmental regulation intensity are constantly improving. As a result, the focus of foreign capital introduction has begun to shift to the central and western regions.

#### 4.1.4. OFDI Regional Differences

**Figure 5:**
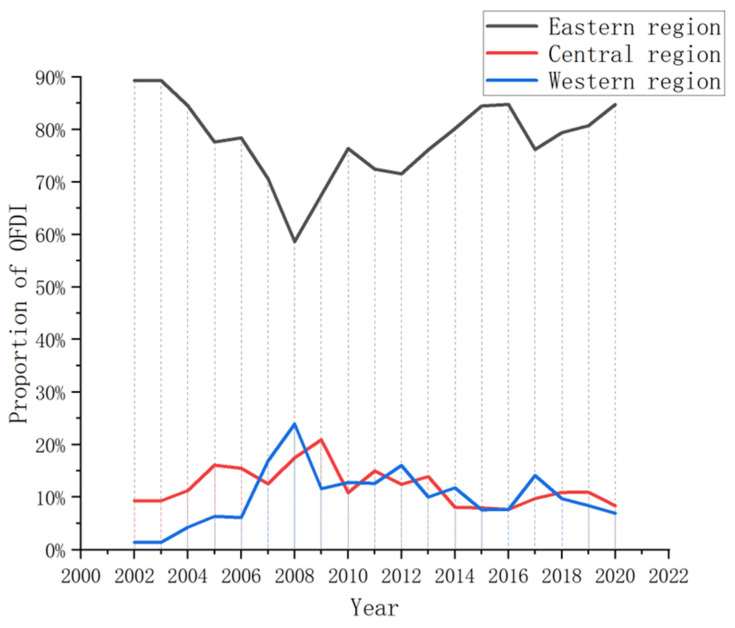
Proportion of OFDI in eastern, central and western China from 2002 to 2020.

In spite of the developments noted in Section 4.1.3, local OFDI in China’s eastern region remains dominant. However, as China’s opening-up strategy continues to extend to inland areas, OFDI in central and western provinces is also gradually increasing, with the gap to coastal areas gradually narrowing. As illustrated in Figure 5, between 2002 and 2019, the proportion of OFDI flow accounted for by the eastern region decreased from 89.31% in 2002 to 80.73% in 2019. Conversely, the central region increased from 9.29% to 10.92%, while the western region increased from 1.40% to 8.38% over the same period. Due to the impact of COVID-19 in 2020, the proportion of OFDI flow in the western region declined by varying degrees. However, its overall development is still rising, indicating that China’s plans for national development, such as the ‘Great Western Development’ strategy, and the rise of the central region policy have achieved remarkable results in promoting the spread of the ‘going global’ strategy to also include enterprises in the central and western regions.

### 4.2. Analysis of the EQI, EPI and ETI

#### 4.2.1. Overall Evolution Trend

**Figure 6:**
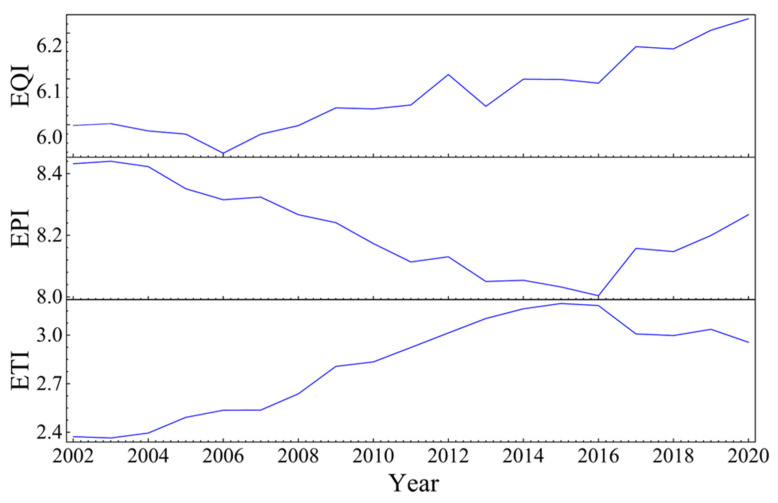
Changes in EQI, EPI and ETI from 2002 to 2020.

As illustrated in Figure 6, the environmental comprehensive quality index demonstrates an upward fluctuation trend during the sample period overall. From 2002 to 2006, EQI decreased gradually, reaching a low value of 5.94. From 2006 to 2020, EQI continued on a gradual upward trend, peaking at 6.23 in 2020. Similarly, the records of the index of environmentally cleaner production over the sample period first decline and then increase. From 2002 to 2015, the EPI exhibited a fluctuating downward trend, peaking at 8.44 in 2003 before falling to 8.03 in 2015 and then gradually rising again in 2020. The environmental terminal governance index first rose and then declined, reaching a low of 2.36 in 2003 and peaking at 3.20 in 2015 before gradually declining once more in 2020. During this time, China strengthened its emphasis on the environment, implemented the ecological civilisation strategy, strengthened environmental protection surrounding construction projects funded by foreign investment and introduced green technologies, leading to a gradual improvement in environmental quality. Environmental regulation measures have also shifted from end-of-pipe treatment to cleaner production, while the move from the ‘pollution end treatment mode’ to the ‘green development mode of cleaner production’ has also begun.

#### 4.2.2. Distribution Map of EQI, EPI and ETI

**Figure 7:**
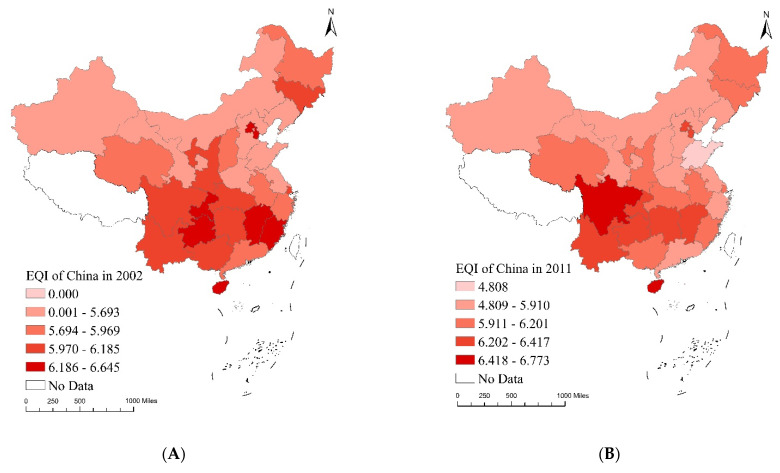

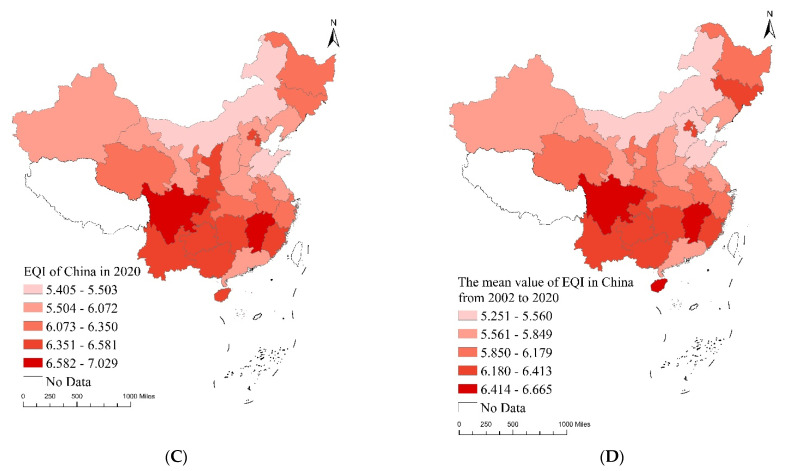
Temporal evolution maps of EQI in China: (**A**) EQI distribution map 2002, (**B**) EQI distribution map 2011, (**C**) EQI distribution map 2020, (**D**) EQI mean value map 2002–2020.

**Figure 8:**
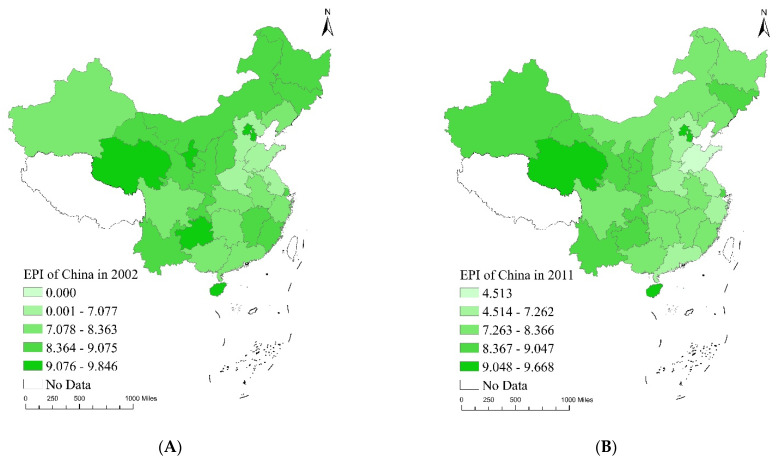

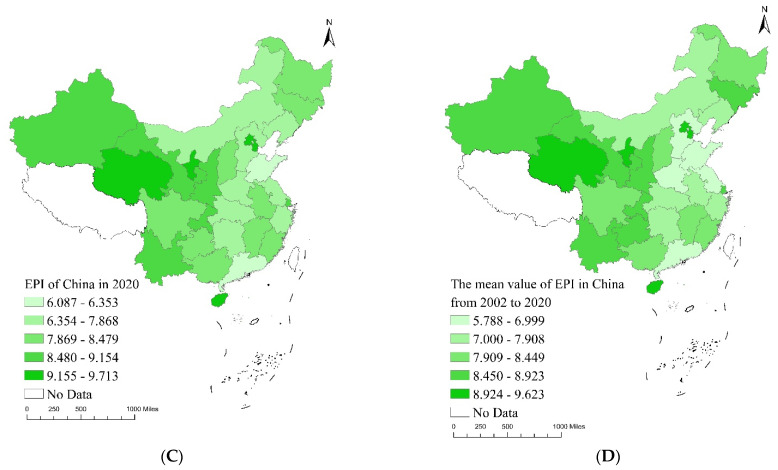
Temporal evolution maps of EPI in China: (**A**) EPI distribution map 2002, (**B**) EPI distribution map 2011, (**C**) EPI distribution map 2020, (**D**) EPI mean value map 2002–2020.

**Figure 9:**
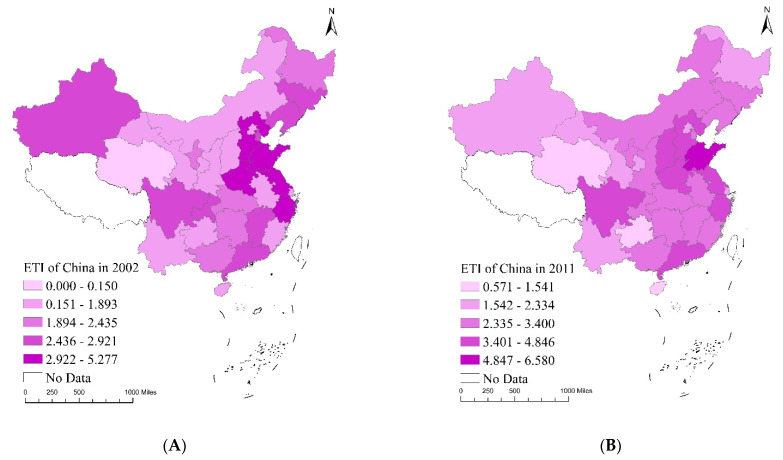

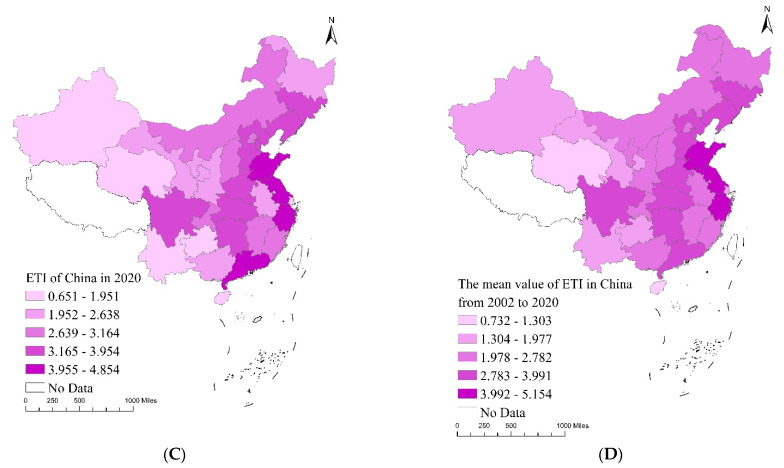
Temporal evolution maps of ETI in China: (**A**) ETI distribution map 2002, (**B**) ETI distribution map 2011, (**C**) ETI distribution map 2020, (**D**) ETI mean value map 2002–2020.

We used ArcGIS 10.C S^®^ to map the distribution of the EQI, EPI and ETI in China over the sample period. Figure 7, Figure 8 and Figure 9 demonstrate the changing trends across these indexes in 2002, 2011 and 2020 and the accompanying mean values. Each index is divided into five categories, from low to high. The darker the red, green and pink shading, the higher the EQI, EPI and ETI in the corresponding years. The EQI, EPI and ETI data were based on manual calculations.

Figure 7 contains temporal evolution maps of the EQI in China. In 2002, the EQI of Hainan province in the eastern region was the highest, benefitting from a high level of ecological endowment and excellent basic environmental conditions. In 2011, The EQI index of Chongqing city in the west and Jiangxi province in the centre began to rise rapidly, mainly because these regions attached great importance to the environment and increased investment in environmental protection as well as economic development. In 2020, the EQI index of Beijing and Tianjin in the east, Sichuan in the west and Hunan in the centre started to increase steadily, mainly because these regions possessed strong economic and technological conditions and were able to improve environmental quality through green development. The average EQI of Chongqing is the largest, indicating its overall environmental strength. During the sample period, the average EQI decreased successively in the order of ‘eastern region (6.00)–central region (6.09)–western region (6.12)’.

Figure 8 contains temporal evolution maps of the EPI in China. In 2002, the EPI index of Qinghai province in the western region was the highest. Due to its small population and minor industrial presence, less pollution is generated there. In 2011, the EPI index of Hainan province, Beijing and Tianjin in the eastern region increased rapidly, benefitting from stronger pollution controls at the source of production. In 2020, the EPI indices of Ningxia and Xinjiang in the west and Shanghai in the east made the greatest progress. These regions possess a solid environmental foundation while also recording the least emissions. Qinghai province has the least pollution emissions based on the average EPI value, indicating that it has achieved the most success in terms of environmentally cleaner production. During the sample period, the average value of the EPI decreased in the order of ‘eastern region (7.92)–central region (8.00)–western region (8.67)’.

Figure 9 contains temporal evolution maps of the ETI in China over the sample period. In 2002, Hebei province in the east and Shandong province in the centre had the highest ETI. This was mainly due to the large number of industrial enterprises operating in these areas, resulting in high pollution emissions in spite of them having the largest amount of pollution control. In 2011, the ETI index of Jiangsu province and Zhejiang province in the east rapidly improved. This can be attributed to the rapid economic development in these areas and the large resultant investment in pollution control. In 2020, the indices of the ETI index of Guangdong province in the east and Chongqing city in the west began to grow rapidly. These regions have strong scientific and technological bases and constantly improve pollution reduction technology. Accountability for pollution has also been strengthened, enabling local enterprises to increase their investment in pollution treatment. The average value of the ETI in Jiangsu province is the highest, indicating its environmental end treatment level is the highest. During the sample period, the average value of the ETI decreased in the order of ‘western region (3.22)–central region (3.00)–eastern region (2.29)’.

## 5. Spatial Difference Analysis

Based on the comprehensive measurement data on China’s environmental quality, we used the Dagum Gini coefficient to quantitatively measure the imbalance and regional gaps in China’s EQI, EPI and ETI and analysed the source of these gaps through coefficient decomposition.

### 5.1. Overall Regional Differences

As illustrated in Table 4, from 2002 to 2020, the average Gini coefficient of China’s EQI was 0.032, demonstrating an overall ‘M’ type trend with a large range of fluctuations, reaching a low of 0.026 in 2005 and a peak of 0.038 in 2016. From 2005 to 2014, the overall Gini coefficient demonstrated a fluctuating upward trend, with an average annual growth rate of 4.347%. From 2016 to 2020, the overall Gini coefficient demonstrated a fluctuating downward trend, with an average annual decline of 4.946%, indicating that regional differences had a slow downward trend, with the overall synergy of China’s EQI enhanced. From 2002 to 2020, the average regional differences between the EPI and ETI were 0.064 and 0.203, respectively, and the overall trend was relatively stable. EPI peaked at 0.072 in 2016 and was at its lowest in 2007 at 0.059. The average annual rate of change during the sample period was 0.038%. The ETI peaked at 0.236 in 2004, recording its lowest value of 0.180 in 2017. The average annual rate of change during the sample period was −1.021%. The overall regional difference in the EQI is smaller than the overall regional difference in the EPI and ETI, and the volatility is extensive. The overall Gini coefficient of the ETI is far greater than the EQI and EPI. Formulating and implementing regional coordinated governance and governance policies are urgently required to effectively narrow the gap between the regional environmental governance levels.

The Dagum Gini coefficient divides the sources of regional disparities into three types: intra-regional differences, inter-regional differences, and hypervariable densities. From the decomposition of the Gini coefficient and the change in the contribution degree of each source, the internal mechanism of the overall regional differences in the EQI, EPI and ETI has changed over time. From 2002 to 2020, the overall Gini coefficient of the EQI demonstrated a distribution pattern of ‘hypervariable density–intra-regional difference–inter-regional difference’, decreasing in turn. Specifically, the average annual variation rate of intra-regional differences was 1.218%, while the average annual contribution rate was 32.70%. The average annual variation rate of inter-regional differences was 26.591%, with an average annual contribution rate of 15.41%. The average annual variation rate of the hypervariable density was −0.696%, and the average annual contribution rate was 51.57%. The overall regional difference in EPI from 2002 to 2020 presents a distribution pattern of ‘hypervariable density–inter-regional difference–intra-regional difference’, decreasing in order. The overall regional difference of the ETI from 2002 to 2020 presents a distribution pattern of ‘inter-regional difference–hypervariable density–intra-regional difference’, decreasing in sequence. The average annual variation rate of intra-regional differences was −1.505%, while the average annual contribution rate was 29.99%. The average annual variation rate of inter-regional differences was −0.023%, with an average annual contribution rate of 38.15%. The average annual variation rate of hypervariable density was −0.768%, and the average annual contribution rate was 31.91%.

These results indicate that, first, hypervariable density is the primary source of the overall regional difference in EQI. The annual average variation rate for regional differences is the largest, indicating that the hypervariable density of regional differences in China’s environmental quality, and the overlapping phenomenon of regional and intra-regional differences are the most obvious, reflecting the weak intra-regional synergy effect. In contrast, the inter-regional difference fluctuates greatly, offering the potential for collaborative spatial governance. Second, hypervariable density is the primary source of overall regional differences in the EPI. The annual average change rate of intra-regional differences, inter-regional differences and hypervariable density is positive, indicating that the spatial difference in cleaner production is expanding, while regional coordination needs to be further improved and major improvements are still required with regard to cleaner production quality and efficiency. Third, the average contribution of inter-regional differences to the overall regional differences in ETI is the largest, and the annual average change rate of intra-regional differences, inter-regional differences and hypervariable density is negative, indicating that the efficiency of environmental governance is low, and the regional disharmony of the ETI inhibits the improvement of regional ecological civilisation construction.

### 5.2. Intra-Regional Differences

As illustrated in Table 5, the intra-regional Gini coefficients of the EQI, EPI and ETI averaged 0.032, 0.064 and 0.203 from 2002 to 2020, respectively, with average annual growth rates of 0.705%, 0.038% and −1.021%. The fluctuation range was small and tended to stabilise over time. This demonstrates that the EQI and EPI exhibit continuous growth overall while the regional gap is expanding, and the regional average difference in the ETI is the largest. This indicates that the spatial difference in environmental governance is the largest, but the regional gap is decreasing.

In terms of regions, the average Gini coefficient in the EQI and ETI demonstrates a distribution pattern of ‘east–west–central’ from 2002 to 2020, decreasing in turn, while the average Gini coefficient in the EPI demonstrates a distribution pattern of ‘east–central–west’. Among them, the average regional Gini coefficients of the EQI, EPI and ETI in the eastern region are 0.038, 0.091 and 0.234, with average annual change rates of −0.126%, 0.491% and −0.618%, respectively. The average regional Gini coefficients of the EQI, EPI and ETI in the central region are 0.022, 0.036 and 0.099, with average annual change rates of 2.975%, −2.397% and −2.152%, respectively, while the average regional Gini coefficients of the EQI, EPI and ETI in the central region are 0.022, 0.036 and 0.099, with average annual change rates of 1.547%, 1.005 and −1.799%, respectively. These findings demonstrate that, first, the regional difference between the EQI and EPI is less than that of the environmental governance index, indicating that cleaner production and environmental governance have not yet formed a mechanism for regional internal coordination and evolution, thus effectively reducing the regional difference in environmental quality. Second, after 2016, the EQI, EPI and ETI Gini coefficients all demonstrate a convergence toward decreasing regional difference fluctuations, indicating that regional coordinated development, cleaner production and end-of-pipe governance promote the improvement of environmental quality in a coordinated manner, thus narrowing the regional environmental quality gap.

### 5.3. Inter-Regional Differences

As illustrated in Table 6, from 2002 to 2020, the average inter-regional Gini coefficient of the EQI, EPI and ETI demonstrates a distribution pattern of ‘east–west, east–central and central–west’, decreasing in turn. The average inter-regional Gini coefficients of the EQI, EPI, and ETI in the eastern central region are 0.033, 0.074 and 0.192, with annual average change rates of 0.420%, −0.143% and −0.989%, respectively. The average intra-regional Gini coefficients of the EQI, EPI and ETI in the eastern–western region are 0.036, 0.078 and 0.262, with annual average change rates of 0.805%, 0.534% and −0.287%, respectively. The average intra-regional Gini coefficients of the EQI, EPI and ETI in the central–western region are 0.027, 0.050 and 0.175, respectively. The average annual change rates are 1.973%, 0.184% and −1.445%, respectively. These findings demonstrate that, first, EQI, EPI and ETI have a large regional gap, and a spatial pattern of coordinated development has not yet been formed. Second, the gap between the east and the central and western regions in terms of EQI, EPI and ETI is still widening. As China’s most densely populated, economically developed and most polluted region, the east must accelerate the coordinated improvement of cleaner production capacity and terminal treatment capacity to achieve sustainable economic development under the existing resource and environmental constraints to improve its sustainable development capacity.

## 6. Empirical Results and Discussion

### 6.1. Simulation Test

#### 6.1.1. Unit-Root Test

This study has adopted the following unit-root tests: the LLC test, IPS test, Fisher ADF test, and Fisher PP test [38]. When at least three methods were passed unanimously, the variable was considered stable. The inspection results are listed in Table 7. The results demonstrate that under the four test methods, the first-order difference components of each variable were stable at a significance level of 1%, so all variables are I (1) processes.

#### 6.1.2. Panel Cointegration Tests

Kao and Pedroni cointegration tests were used to determine whether a long-term cointegration relationship existed between variables. The test results are listed in Table 8. The results demonstrate that the cointegration test negates the original hypothesis at the 1% significance level, indicating a cointegration relationship between the model’s variables, and that regression analyses can be conducted.

### 6.2. Dynamic Panel Regression Analysis

#### 6.2.1. Overall National Regression Analysis

Before the regression analysis, Hausmann’s test was conducted on the panel data. The test resulted in a *p*-value of 0.000, which strongly rejects the null hypothesis and indicates the use of fixed effects. To reduce the dynamic panel error as much as possible, the SYS-GMM estimation method was adopted to estimate the dynamic panel model [39]. As illustrated in Table 9, columns (1), (3), (5), (2), (4) and (6) demonstrate the estimated results of SYS-GMM and the fixed effects model, respectively. The last two lines of the table show the *p*-values of the AR (1) and AR (2) tests.

Table 9 illustrates the regression results of the dynamic SYS-GMM and the static, fixed effects. In general, the coefficient symbols of all variables did not change under the two estimation methods, proving the robustness of the regression results. In addition, the lags of the comprehensive EQI, EPI and environmental end-of-pipe governance index by one period have a significant positive impact on the current period, indicating that China’s EQI, EPI and ETI have dynamic sustainability. Given that the test results in this study have accepted the original hypothesis of AR (1) and rejected the original hypothesis of AR (2) and there was no second-order or higher-order autocorrelation, we selected the estimation results of the one-step GMM for analysis.

The estimation results demonstrate that lnIFDI significantly positively impacted environmental quality and cleaner production, with coefficients of 0.431 and 0.332, respectively, passing at least a 10% robustness test. LnIFDI had a negative impact on the environmental end treatment, with a coefficient of −0.035, but this was not significant. LnOFDI significantly promotes lnEQI, lnEPI and lnETI, with coefficients of 0.112, 0.232 and 0.137, respectively, passing at least a 10% significance test. This demonstrates that through two-way FDI, China has absorbed advanced environmental protection technologies, improved the efficiency of cleaner production, transferred excess capacity, and generally improved environmental quality. Ln(IFDI × OFDI) has a positive promotional effect on lnEQI and lnEPI but a negative effect on lnETI. This also indicates that the mutual adjustment of IFDI and OFDI has promoted comprehensive environmental governance and cleaner production levels but has hindered environmental end treatment to a certain extent. This reflects the concept of strong, sustainable development and the transition from the extensive development of pollution before treatment to the green development of cleaner production.

The control variables demonstrate that lnED and ES have a negative impact on lnEQI and lnEPI and a significant positive impact on lnETI. IND can promote lnEQI and lnEPI and inhibit lnETI. ER and RD have a positive impact on environmental quality, environmentally cleaner production and environmental end treatment. LnPOP inhibits lnEQI, lnEPI and lnETI.

#### 6.2.2. Regional Regression Analysis

To further explore the impact of various factors on different areas, we used SYS-GMM to estimate the impact of various factors. As illustrated in Table 10, columns (1), (2), and (3) demonstrate the regression results for the comprehensive EQI, EPI and ETI in the eastern region, and columns (4), (5), (6), (7), (8), and (9) demonstrate the corresponding estimation results in the central and western regions. The last two lines of the table are the *p*-values of the AR (1) and AR (2) tests.

In the regression results shown in Table 9, the EQI, EPI and ETI in the three major regions of eastern, central and western China lagged by one period and had a significant positive impact on the current period, all of which passed the 1% significance test, indicating that they have dynamic sustainability. Therefore, the one-step GMM estimation results were selected for the analysis.

In the eastern region, LnIFDI had a significant positive impact on lnEQI and pro-moted the increase in lnEPI, but it was not significant. It also had a negative impact on lnETI, but not significantly. LnOFDI can promote lnEQI, but this effect was not significant. It had a significant positive impact on lnEPI and lnETI and passed the 10% significance test. Ln(IFDI × OFDI) had a positive impact on environmental quality and environmental end-of-pipe governance and a negative effect on lnETI, but this effect was not significant, indicating that the interaction between IFDI and OFDI in the eastern region further optimises the environmental benefits of FDI by increasing the level of foreign investment. LnED had a negative impact on lnEQI and lnEPI and a positive impact on lnETI. IND can promote lnEQI and lnEPI and hinder lnETI. ES and lnPOP negatively impacted lnEQI, lnEPI and lnETI, while ER and RD positively impacted the overall quality of the environment, environmentally cleaner production and environmental end treatment.

In the central region, LnIFDI promoted lnEQI and lnEPI and passed at least a 10% significance test. It hindered lnETI; however, the impact was not significant. LnOFDI had a significant positive impact on lnEQI and lnETI, passed the 10% significance test, and had a negative effect on lnEPI. Ln(IFDI × OFDI) had a positive impact on lnEQI and lnEPI, indicating that IFDI and OFDI have a mutual regulatory effect, further promoting the improvement of environmental quality and the level of cleaner production in the central region. It negatively impacts lnETI, indicating that the increase in OFDI in the central region leads to further deterioration of the IFDI’s ETI. LnED has a negative impact on lnEQI and lnEPI and a positive impact on lnETI. IND can promote lnEQI and lnEPI and hinder lnETI. ES negatively impacts lnEQI and lnEPI and has a positive impact on lnETI. ER and RD have a positive impact on the comprehensive quality of the environment, environmentally cleaner production and environmental end treatment. However, lnPOP has a negative impact on lnEQI, lnEPI and lnETI.

In the western region, lnIFDI significantly promoted lnEQI and lnEPI, passing at least a 5% significance test, but significantly hindered lnETI, passing the 5% significance test. LnOFDI had a significant positive impact on lnEQI and lnETI, passing at least a 10% significance test, and a significant negative impact on lnEPI, passing the 1% significance test. Ln(IFDI*OFDI) has a positive impact on lnEQI, which indicates that the interaction between IFDI and OFDI further promotes the improvement of environmental quality in the western region, while it negatively impacts lnEPI and lnETI, indicating that the interaction between IFDI and OFDI in the western region significantly reduces environmentally cleaner production and environmental end treatment. LnED and ES had negative effects on lnEQI and lnEPI and positive effects on lnETI. IND can promote lnEQI and lnEPI and hinder lnETI. ER and RD have a positive impact on the comprehensive quality of the environment, environmentally cleaner production and environmental end treatment, while lnPOP negatively impacts lnEQI, lnEPI and lnETI.

### 6.3. Discussion

First, it must be stated that two-way FDI plays a great role in promoting China’s environmental quality. IFDI has brought not only funds but has also introduced advanced environmental protection technology, which has promoted the upgrading and development of China’s environmental protection industry. The advanced environmental protection standards and management experience of multinational companies have played a vital learning and demonstration role and produced a diffusion effect, prompting local enterprises to strengthen ‘green production’ and ‘green procurement’, guiding consumers to ‘green consumption’ and improving environmental awareness. In addition, Chinese enterprises can use the OFDI reverse technology spillover to improve their production technology and management experience, reduce energy consumption and improve their ability to treat industrial waste in order to improve their level of environmental pollution.

However, two-way FDI has also had an evidently negative impact on China’s environmental quality. About 30% of IFDI flows into China’s pollution-intensive industries, such as paper and paper products, leather, and fur, alongside other industries. Because China carries out export trade at a relatively low cost and implements tax rebates and other fiscal policies to encourage exports, the benefits of export trade are shared both at home and abroad. However, the pollution remains at home and is borne by China alone. The rapid growth of OFDI has also accelerated the export of domestic goods. Many enterprises lack scientific and long-term investigation and planning prior to investment, and so they rush to start projects, which not only causes losses to the enterprises but also leads to domestic resource depletion and increased environmental pollution.

Second, according to the regression results, FDI significantly impacts environmental quality and cleaner production at the national level. Although it negatively impacts the environmental end-of-pipe treatment, the estimated results have not passed the significance test, while China is gradually transforming from an extensive production model to a strong, sustainable, cleaner production model. The eastern and central regions should fully learn from the clean technology and management experience provided by FDI while strengthening the improvement in the IFDI environmental technology spillover effect on the production process to, in turn, improve the clean production technology of local enterprises and accelerate the introduction of new industries with low energy consumption and high added value. The inflow of IFDI to the western region has a significant positive effect on improving its environmental terminal governance capacity. With the proposal of the ‘Belt and Road’ initiative, China’s western region will absorb more FDI. The western region should firmly understand the advantages of preferential policies and regional locations. It should focus on selectively introducing high-quality FDI with low energy consumption and high added value, combining it with characteristic local industries and resource endowments. Full prioritisation should be given to the technology spillover effect of IFDI, fully learning from the clean technology and management experience of FDI and promoting the improvement of ‘clean production’ and pollution control capacity [40].

Third, regarding OFDI, China should seek to actively promote the construction of the ‘Belt and Road’ regional value chain and the international industrial chain. A strong focus should be placed on promoting the transfer of domestic excess capacity to other regions and strengthening international capacity cooperation. Combined development should also be promoted from commodity exports to exports of goods, services, capital and technology to reduce the negative environmental effects caused by the scale effect of FDI. Finally, OFDI can actively encourage and guide domestic direct investment in high-tech industries and obtain reverse technology spillover effects through mergers and acquisitions of overseas high-quality company assets, thus promoting the positive impact of OFDI on environmental quality [41].

Fourth, China should optimise the reward and punishment mechanism, levy a ‘pollution tax’ on foreign-funded enterprises that cause pollution, and provide policy support, tax credits and other policy incentives for foreign-funded projects that are environmentally friendly and cleaner. Improving innovation ability is the key to fully capitalising on the positive environmental effect of two-way FDI synergy. Therefore, the Chinese government should use tax relief, innovation subsidies and other policy means to encourage enterprises to improve their technological innovation ability, apply cleaner production technology and accelerate the penetration of technological innovation in the ecological field [42].

## 7. Conclusions

The report of the 20th National Congress of the CPC has proposed to ‘promote green development and achieve a high-level opening-up’ based on strong, sustainable development. This study has constructed a comprehensive evaluation index system of environmental quality from the perspective of environmentally cleaner production and environmental end treatment. EQI, EPI and ETI have been calculated for China’s provinces from 2002 to 2020. ArcGIS and the Dagum Gini coefficient were then used to analyse the temporal evolution and spatial differences of the EQI, EPI and ETI, and a dynamic GMM and fixed-effect model was adopted to study the impact of IFDI and OFDI on environmental quality.

First, the proportion of IFDI and OFDI is predominant in the eastern region, but the gap with the central and western regions is gradually narrowing. During the sample period, the EQI curve demonstrated an upward trend of fluctuation, and the average value of the EQI was ‘eastern region–central region–western region’, in decreasing order. The curve of the EPI had a ‘U’ shape, which first decreased and then rose. The EPI’s average value decreased in the order of ‘eastern region–central region–western region’. The curve of the ETI had an inverted ‘U’ shape that first rose and then fell, and the average value of the ETI decreased successively in the order of ‘western region–central region–eastern region’.

Second, for overall spatial differences represented by the Dagum Gini coefficient, from 2002 to 2020, the EQI demonstrated a distribution pattern of ‘hypervariable density–intra-regional difference–inter-regional difference’, decreasing in turn. The EPI demonstrated a distribution pattern of ‘hypervariable density–inter-regional difference–intra-regional difference’, decreasing in sequence. The ETI presented a distribution pattern of ‘inter-regional difference–hypervariable density–intra-regional difference’, decreasing in sequence. For intra-regional differences, the average Gini coefficient in the EQI and ETI demonstrated a distribution pattern of ‘east–west–central’, decreasing in turn, while the average Gini coefficient in the EPI demonstrated a distribution pattern of ‘east–central –west’, decreasing in turn. For inter-regional differences, the average inter-regional Gini coefficient of the EQI, EPI and ETI demonstrated a distribution pattern of ‘east–west, east–central and central–west’, decreasing in turn.

Finally, from the estimation results, lnIFDI had a significant positive impact on environmental quality and cleaner production, with coefficients of 0.431 and 0.332, respectively, which had a negative impact on environmental end treatment, with a coefficient of −0.035. LnOFDI significantly promoted lnEQI, lnEPI and lnETI, with coefficients of 0.112, 0.232 and 0.137, respectively, passing at least a 10% significance test. This demonstrates that through two-way FDI, China has improved cleaner production efficiency and environmental quality generally. lnIFDI has promoted the improvement of lnEQI and lnEPI in the eastern, central and western regions but has hindered lnETI. At the same time, lnOFDI has positively impacted the environmental effects in the eastern and central regions, while it has promoted lnEQI and lnETI and hindered EPI in the western region.

This study provides the following policy recommendations. First, China should establish a stricter environmental access system, encourage foreign enterprises to introduce advanced cleaner production technologies, and seek to improve the cleaner production efficiency of high-polluting industries through the technology diffusion effect of IFDI. Second, China should increase its efforts to ‘go global’. Governments at all levels should encourage state-owned and private enterprises to participate in OFDI, which is not only conducive to the enhancement of production capacity but can also enable the learning of advanced green technologies and management concepts through the international market. Third, China should actively participate in multilateral cooperation in environmental governance through two-way FDI, such as atmospheric governance and desert governance. Domestic enterprises should strengthen cooperation and exchange, provide each other with useful knowledge based on experience and strengthen the response to climate change while striving to achieve the goal of ‘carbon peak and carbon neutral’ and jointly maintaining the sustainable development of the world [43]. This study has several limitations. Owing to data limitations, the impact of two-way FDI on environmental quality was assessed at the provincial level. However, cities and counties are often the main attractions for investment. In the future, an analysis will be carried out on the environmental effects of regional foreign trade at the municipal and county levels to make the research more in-depth. Future research can also seek to combine pollution data, technology input and output data at the enterprise level to discuss the performance of the parent company, domestic subsidiaries and foreign subsidiaries in terms of pollution emissions as well as the adaptation of cleaner production and end-of-pipe treatment to provide a reference point for targeted environmental regulation.

## Figures and Tables

**Table 1:** China’s comprehensive environmental quality assessment index system.

Target	Dimension	Criterion	Specific Index	Unit	Attribute
Comprehensive environmental quality index (EQI)	Environmentally cleaner production index (EPI)	Industrial environmental pollution production	Industrial wastewater production	10,000 tons	−
			Industrial waste gas generation	10,000 tons	−
			Industrial smoke and dust production	10,000 tons	−
			Industrial dust production	10,000 tons	−
			Sulphur dioxide production	10,000 tons	−
			Production of industrial chemical oxygen demand	10,000 tons	−
			Industrial ammonia nitrogen production	10,000 tons	−
			Output of industrial solid waste	10,000 tons	−
			Production of industrial hazardous waste	10,000 tons	−
		Domestic environmental pollution production	Domestic waste production	10,000 tons	−
			Output of domestic wastewater	10,000 tons	−
			Area of environmental noise	km^2^	−
			Carbon dioxide production	10,000 tons	−
		Agricultural environmental pollution production	Fertiliser application amount	ton	−
			Pesticide usage	ton	−
			Area of soil erosion	km^2^	−
			Land desertification area	km^2^	−
	Environmental end treatment index (ETI)	Industrial pollution treatment	Industrial wastewater treatment amount	10,000 tons	+
			Treatment amount of industrial smoke and dust	10,000 tons	+
			Industrial dust control amount	10,000 tons	+
			SO_2_ treatment amount	10,000 tons	+
			Chemical oxygen demand treatment amount	10,000 tons	+
			Ammonia nitrogen treatment amount	10,000 tons	+
			Solid waste treatment amount	10,000 tons	+
			Treatment amount of hazardous waste	10,000 tons	+
		Domestic pollution treatment	Domestic waste treatment amount	10,000 tons	+
			Domestic sewage treatment amount	10,000 tons	+
			Noise pollution control area	km^2^	+
			Construction area of high pollution, no burning area	km^2^	+
		Agricultural pollution treatment	Soil erosion control area	km^2^	+
			Land desertification control area	km^2^	+
			Input intensity of agricultural environmental governance	%	+

Note: The environmental pollution production was reversed according to the evaluation method cited above. The larger the EPI index, the stronger the environmentally cleaner production capacity. In the attribute, ‘+’ indicated that the indicator is positive, and ‘−’ indicated that the indicator is negative.

**Table 2:** Definitions of variables and data sources.

Variable	Definition	Source
EQI	Provincial comprehensive environmental quality index	Calculation
EPI	Provincial environmentally cleaner production index	Calculation
ETI	Provincial environmental end treatment index	Calculation
IFDI	Provincial IFDI flow	Wind
OFDI	Provincial OFDI flow	Wind
IFDI × OFDI	Interaction between IFDI and OFDI	Wind
ED	Economic development level: provinces’ GDP/population of each province	CSY
IND	Industrial structure: provinces’ tertiary sector value added/provinces’ GDP	CSY
ES	Energy structure: Proportion of coal in energy consumption of each province	PSY
ER	Environmental regulation: provinces’ pollution treatment cost/provinces’ added value of secondary industry	PSY
RD	Research and development investment: provinces’ R&D investment/provinces’ GDP	PSY
POP	Population density: population of each province/total provinces’ area	CSY

Notes: Calculation: calculated by the vertical and horizontal scatter grade method; CSY: China Statistical Yearbook. PSY: Provincial Statistical Yearbook. Wind: financial database. For the data used in this study, the timeframe was 2002–2020.

**Table 3:** Descriptive statistics of variables.

Variable	Obs	Mean	Std. Dev.	Min	Max	Source
lnEQI	570	1.8005	0.0604	1.5674	1.9533	Calculation
lnEPI	570	2.0985	0.1277	1.4951	2.2882	Calculation
lnETI	570	0.9486	0.4583	−1.8958	1.8841	Calculation
lnIFDI	570	38.446	1.6902	10.8277	16.7781	Wind
lnOFDI	570	0.021	2.7843	3.7376	16.6355	Wind
ln(IFDI × OFDI)	570	0.021	4.0205	16.3218	33.2449	Wind
lnED	570	1.5864	0.7072	0.5853	3.8010	CSY
IND	570	0.4401	0.0914	0.3278	0.8352	CSY
ES	570	0.4367	0.1591	0.0071	0.8689	PSY
ER	570	0.0035	0.0031	0.0001	0.0245	PSY
RD	570	0.0177	0.0185	0.0001	0.0642	PSY
lnPOP	570	0.8223	1.2341	−2.5910	3.3824	CSY

Notes: Calculation: calculated by the vertical and horizontal scatter grade method; CSY: China Statistical Yearbook. PSY: Provincial Statistical Yearbook. Wind: financial database. For the data used in this study, the timeframe was 2002–2020.

**Table 4:** Gini coefficients and their decomposition terms of the EQI, EPI and ETI in China from 2002 to 2020.

Year	EQI				EPI				ETI			
	G	G_w_	G_nb_	G_t_	G	G_w_	G_nb_	G_t_	G	G_w_	G_nb_	G_t_
2002	0.028	0.009	0.001	0.019	0.061	0.019	0.019	0.023	0.235	0.073	0.092	0.071
2003	0.030	0.010	0.001	0.019	0.061	0.019	0.019	0.023	0.235	0.072	0.093	0.069
2004	0.028	0.009	0.002	0.016	0.060	0.019	0.019	0.023	0.236	0.072	0.093	0.071
2005	0.026	0.009	0.002	0.016	0.061	0.017	0.018	0.026	0.219	0.067	0.073	0.079
2006	0.029	0.009	0.005	0.014	0.061	0.018	0.023	0.020	0.208	0.062	0.083	0.063
2007	0.028	0.009	0.003	0.016	0.059	0.018	0.019	0.022	0.211	0.063	0.086	0.062
2008	0.031	0.010	0.005	0.016	0.067	0.020	0.027	0.020	0.230	0.069	0.100	0.061
2009	0.032	0.011	0.006	0.015	0.064	0.019	0.023	0.022	0.206	0.064	0.074	0.068
2010	0.031	0.011	0.007	0.014	0.065	0.019	0.024	0.022	0.210	0.065	0.081	0.064
2011	0.033	0.011	0.007	0.015	0.068	0.020	0.025	0.023	0.205	0.063	0.073	0.069
2012	0.033	0.011	0.007	0.016	0.064	0.019	0.022	0.023	0.188	0.056	0.076	0.056
2013	0.035	0.012	0.005	0.018	0.070	0.021	0.022	0.027	0.190	0.058	0.067	0.065
2014	0.038	0.012	0.006	0.020	0.068	0.020	0.021	0.027	0.185	0.055	0.070	0.061
2015	0.035	0.012	0.006	0.017	0.068	0.020	0.022	0.026	0.182	0.054	0.064	0.064
2016	0.038	0.013	0.008	0.018	0.072	0.021	0.023	0.028	0.187	0.057	0.064	0.066
2017	0.035	0.012	0.008	0.016	0.066	0.019	0.021	0.025	0.180	0.053	0.063	0.065
2018	0.032	0.011	0.005	0.017	0.063	0.015	0.023	0.025	0.187	0.053	0.071	0.063
2019	0.031	0.010	0.006	0.015	0.062	0.015	0.022	0.025	0.181	0.050	0.071	0.059
2020	0.031	0.010	0.005	0.016	0.060	0.018	0.020	0.022	0.191	0.054	0.081	0.057
mean value	0.032	0.010	0.005	0.016	0.064	0.019	0.022	0.024	0.203	0.061	0.078	0.065

Source: Calculation and arrangement of this paper.

**Table 5:** Intra-regional differences in Gini coefficients of the EQI, EPI and ETI in China from 2002 to 2020.

Year	EQI				EPI				ETI			
	All	East	Central	West	All	East	Central	West	All	East	Central	West
2002	0.028	0.035	0.020	0.025	0.061	0.082	0.041	0.038	0.235	0.233	0.158	0.230
2003	0.030	0.035	0.024	0.025	0.061	0.081	0.045	0.039	0.235	0.235	0.159	0.221
2004	0.028	0.035	0.023	0.022	0.060	0.080	0.045	0.037	0.236	0.234	0.164	0.220
2005	0.026	0.033	0.018	0.023	0.061	0.076	0.046	0.041	0.219	0.233	0.167	0.169
2006	0.029	0.038	0.015	0.023	0.061	0.082	0.035	0.034	0.208	0.224	0.117	0.164
2007	0.028	0.036	0.021	0.021	0.059	0.077	0.038	0.036	0.211	0.223	0.120	0.171
2008	0.031	0.043	0.019	0.023	0.067	0.104	0.033	0.033	0.230	0.266	0.115	0.166
2009	0.032	0.043	0.019	0.028	0.064	0.099	0.036	0.027	0.206	0.258	0.098	0.159
2010	0.031	0.041	0.017	0.029	0.065	0.098	0.037	0.029	0.210	0.258	0.091	0.162
2011	0.033	0.044	0.019	0.028	0.068	0.104	0.034	0.028	0.205	0.255	0.099	0.154
2012	0.033	0.039	0.026	0.029	0.064	0.095	0.037	0.027	0.188	0.222	0.078	0.148
2013	0.035	0.043	0.023	0.030	0.070	0.104	0.039	0.031	0.190	0.241	0.067	0.146
2014	0.038	0.044	0.028	0.036	0.068	0.103	0.037	0.032	0.185	0.236	0.061	0.129
2015	0.035	0.041	0.027	0.032	0.068	0.101	0.040	0.030	0.182	0.240	0.063	0.121
2016	0.038	0.045	0.026	0.037	0.072	0.104	0.037	0.035	0.187	0.242	0.076	0.131
2017	0.035	0.036	0.026	0.035	0.066	0.090	0.025	0.039	0.180	0.220	0.055	0.142
2018	0.032	0.032	0.023	0.035	0.063	0.081	0.024	0.045	0.187	0.216	0.056	0.153
2019	0.031	0.031	0.024	0.032	0.062	0.084	0.028	0.040	0.181	0.206	0.050	0.141
2020	0.031	0.031	0.026	0.031	0.060	0.083	0.023	0.041	0.191	0.201	0.079	0.156
mean value	0.032	0.038	0.022	0.029	0.064	0.091	0.036	0.035	0.203	0.234	0.099	0.162

Source: Calculation and arrangement of this paper.

**Table 6:** Inter-regional differences in Gini coefficients of the EQI, EPI and ETI in China from 2002 to 2020.

Year	EQI			EPI			ETI		
	E-C	E-W	C-W	E-C	E-W	C-W	E-C	E-W	C-W
2002	0.030	0.031	0.024	0.070	0.071	0.050	0.219	0.284	0.221
2003	0.031	0.027	0.031	0.070	0.070	0.050	0.218	0.286	0.220
2004	0.031	0.030	0.024	0.069	0.069	0.049	0.222	0.285	0.223
2005	0.028	0.029	0.021	0.068	0.069	0.053	0.211	0.259	0.210
2006	0.031	0.034	0.021	0.069	0.075	0.047	0.198	0.270	0.178
2007	0.030	0.031	0.023	0.066	0.071	0.048	0.198	0.274	0.184
2008	0.034	0.036	0.023	0.080	0.086	0.044	0.224	0.300	0.181
2009	0.033	0.038	0.025	0.077	0.079	0.045	0.199	0.261	0.167
2010	0.031	0.037	0.024	0.076	0.080	0.047	0.203	0.272	0.164
2011	0.035	0.039	0.025	0.081	0.084	0.049	0.197	0.259	0.169
2012	0.036	0.036	0.029	0.077	0.080	0.044	0.182	0.246	0.151
2013	0.036	0.040	0.028	0.084	0.085	0.051	0.184	0.250	0.150
2014	0.039	0.042	0.033	0.082	0.084	0.048	0.178	0.246	0.152
2015	0.036	0.039	0.030	0.081	0.084	0.051	0.179	0.240	0.146
2016	0.037	0.043	0.033	0.084	0.087	0.055	0.184	0.242	0.148
2017	0.033	0.039	0.032	0.073	0.081	0.055	0.165	0.240	0.157
2018	0.029	0.036	0.030	0.065	0.075	0.061	0.162	0.256	0.175
2019	0.030	0.034	0.030	0.067	0.075	0.055	0.158	0.251	0.168
2020	0.030	0.034	0.030	0.065	0.075	0.049	0.176	0.262	0.165
mean value	0.033	0.036	0.027	0.074	0.078	0.050	0.192	0.262	0.175

Source: Calculation and arrangement of this study. E-C represents east–central, E-W represents east–west, and C-W represents central–west.

**Table 7:** Unit-root test.

Variables	Order	LLC (*p*-Value)	IPS (*p*-Value)	ADF (*p*-Value)	PP (*p*-Value)
lnEQI	I (0)	0.238	0.001	0.030	0.000
	I (1)	0.000	0.000	0.000	0.000
lnEPI	I (0)	0.170	0.618	0.910	1.000
	I (1)	0.000	0.000	0.000	0.000
lnETI	I (0)	0.200	0.491	0.605	1.000
	I (1)	0.000	0.000	0.000	0.000
lnIFDI	I (0)	1.000	1.000	0.980	0.561
	I (1)	0.008	0.000	0.000	0.000
lnOFDI	I (0)	0.060	1.000	1.000	0.901
	I (1)	0.000	0.000	0.000	0.000
ln(IFDI × OFDI)	I (0)	0.590	0.402	0.222	0.709
	I (1)	0.000	0.000	0.000	0.000
lnED	I (0)	0.080	0.263	0.000	0.440
	I (1)	0.000	0.000	0.000	0.000
IND	I (0)	1.000	0.990	0.912	1.000
	I (1)	0.000	0.000	0.000	0.000
ES	I (0)	0.100	0.130	0.010	0.215
	I (1)	0.000	0.000	0.001	0.000
ER	I (0)	0.130	0.060	0.930	0.990
	I (1)	0.000	0.000	0.000	0.003
RD	I (0)	0.271	0.009	0.330	0.570
	I (1)	0.000	0.000	0.000	0.003
lnPOP	I (0)	0.031	0.371	0.620	1.000
	I (1)	0.000	0.000	0.030	0.000

**Table 8:** Panel cointegration test.

Testing Method	Testing Type	Statistic Values (*p*-Value)
Kao test	ADF	−10.5755 *** (0.000)
Pedroni test	Panel-ADF	−9.8224 *** (0.000)
	Group-ADF	−14.3962 *** (0.000)

Notes: *** are significant at 1% levels, respectively.

**Table 9:** Regression results of influencing factors for EQI, EPI and ETI in China.

Variables	LnEQI		LnEPI		LnETI		
	SYS-GMM	FE	SYS-GMM	FE		SYS-GMM	FE
	(1)	(2)	(3)	(4)		(5)	(6)
L1_lnEQI	1.136 ***	/	/	/		/	/
	(0.212)						
L1_lnEPI	/	/	1.125 ***	/		/	/
			(0.325)				
L1_lnETI	/	/	/	/		0.973 ***	/
						(0.148)	
lnIFDI	0.431 ***	0.449 ***	0.332 *	0.393 *		−0.035	−0.068
	(0.114)	(0.124)	(0.172)	(0.234)		(0.073)	(0.060)
lnOFDI	0.112 *	0.124 *	0.232 *	0.270 **		0.137 **	0.146 *
	(0.064)	(0.067)	(0.122)	(0.134)		(0.067)	(0.078)
ln(IFDI × OFDI)	0.052	0.081 ***	0.039 ***	0.056 *		−0.041	−0.009
	(0.043)	(0.012)	(0.012)	(0.028)		(0.047)	(0.006)
lnED	−0.481 *	−0.473 ***	−0.565 **	−0.493 **		0.684 ***	0.162 *
	(0.264)	(0.142)	(0.282)	(0.213)		(0.216)	(0.090)
IND	0.073	0.006	0.271 ***	0.195 ***		−0.091 **	−0.057 **
	(0.060)	(0.035)	(0.065)	(0.053)		(0.039)	(0.023)
ES	−0.052	−0.092	−0.010	−0.158 *		0.400 ***	0.179 ***
	(0.075)	(0.060)	(0.082)	(0.089)		(0.065)	(0.038)
ER	0.142 ***	0.051 **	0.056	0.028		0.199 ***	0.117 ***
	(0.042)	(0.023)	(0.041)	(0.036)		(0.029)	(0.015)
RD	0.420 **	0.671 **	0.603 ***	0.756 ***		0.483 *	0.558 **
	(0.201)	(0.324)	(0.146)	(0.234)		(0.256)	(0.278)
lnPOP	−0.386 ***	−0.129 **	−0.282 ***	−0.139		−0.311 ***	−0.081 **
	(0.069)	(0.056)	(0.065)	(0.085)		(0.045)	(0.036)
Cons	0.638 ***	0.444 ***	1.006 ***	0.762 ***		0.294 ***	0.563 ***
	(0.046)	(0.053)	(0.048)	(0.080)		(0.030)	(0.034)
Obs	540	540	540	540		540	540
Adj R^2^	/	0.452	/	0.482		/	0.527
AR (1)	0.013		0.022			0.012	
AR (2)	0.279		0.658			0.455	

Notes: *, **, *** are significant at 10%, 5%, and 1% levels, respectively. Standard errors are in parentheses.

**Table 10:** Regression results of influencing factors for EQI, EPI and ETI in the eastern, central and western regions.

	Eastern Region			Central Region			Western Region		
Variables	LnEQI	LnEPI	LnETI	LnEQI	LnEPI	LnETI	LnEQI	LnEPI	LnETI
	(1)	(2)	(3)	(4)	(5)	(6)	(7)	(8)	(9)
L1_lnEQI	1.268 ***	/	/	1.157 ***	/	/	1.138 ***	/	/
	(0.353)			(0.265)			(0.224)		
L1_lnEPI	/	1.024 ***	/	/	1.002 **	/	/	1.024 ***	/
		(0.303)			(0.437)			(0.303)	
L1_lnETI	/	/	0.971 ***	/		0.900 ***	/	/	0.859 ***
			(0.254)			(0.338)			(0.202)
lnIFDI	0.382 *	0.411	−0.280	0.278 **	0.247 *	−0.279	0.482 ***	0.552 **	−0.318 **
	(0.198)	(0.285)	(0.226)	(0.132)	(0.129)	(0.215)	(0.156)	(0.222)	(0.157)
lnOFDI	0.159	0.210 ***	0.121 *	0.171 **	0.349 ***	0.270 ***	0.172 *	−0.369 ***	0.348 ***
	(0.136)	(0.059)	(0.069)	(0.079)	(0.086)	(0.070)	(0.092)	(0.053)	(0.070)
ln(IFDI × OFDI)	0.091 ***	0.016 *	−0.067	0.125 ***	0.037	−0.031 *	0.065	−0.058 **	−0.085
	(0.029)	(0.009)	(0.222)	(0.035)	(0.061)	(0.017)	(0.057)	(0.026)	(0.207)
lnED	−0.735 **	−0.640	0.223 **	−0.523 ***	−0.186	0.172 **	−0.448 *	−0.116	0.122
	(0.308)	(0.646)	(0.111)	(0.181)	(0.154)	(0.088)	(0.235)	(0.215)	(0.167)
IND	0.045	0.053	−0.051	0.025 **	0.081 ***	−0.093	0.156 *	0.072 ***	−0.152 ***
	(0.060)	(0.065)	(0.045)	(0.009)	(0.028)	(0.075)	(0.092)	(0.024)	(0.054)
ES	−0.116	−0.103	−0.160 **	−0.203	−0.170	0.158 ***	−0.261 **	−0.272 ***	0.127 **
	(0.090)	(0.097)	(0.068)	(0.194)	(0.259)	(0.051)	(0.109)	(0.082)	(0.064)
ER	0.024	0.068 *	0.146 ***	0.033	0.015	0.080 ***	0.160 ***	0.041	0.121 ***
	(0.037)	(0.039)	(0.028)	(0.041)	(0.063)	(0.021)	(0.053)	(0.039)	(0.030)
RD	0.459 **	0.556 *	0.109	0.318 ***	0.618 ***	0.261 *	0.195 **	0.621 ***	0.214 **
	(0.222)	(0.317)	(0.141)	(0.106)	(0.185)	(0.145)	(0.068)	(0.117)	(0.091)
lnPOP	−0.006	−0.009	−0.078 *	−0.018	−0.057	−0.059	−0.059	−0.210***	−0.061
	(0.055)	(0.041)	(0.043)	(0.106)	(0.046)	(0.136)	(0.136)	(0.059)	(0.045)
Cons	0.557 ***	1.157 ***	0.493 ***	0.305 ***	0.881 ***	0.610 ***	0.517 ***	1.051 ***	0.549 ***
	(0.054)	(0.058)	(0.041)	(0.105)	(0.238)	(0.103)	(0.136)	(0.103)	(0.080)
Obs	198	198	198	144	144	144	198	198	198
AR (1)	0.012	0.023	0.005	0.018	0.011	0.007	0.013	0.015	0.023
AR (2)	0.283	0.740	0.329	0.477	0.859	0.566	0.158	0.301	0.683

Notes: *, ** and *** are significant at the 10%, 5% and 1% levels, respectively. Standard errors are in parentheses.

## Data Availability

The data presented in this study are available on request from the corresponding author.

## Abbreviations

FDI	Two-way foreign direct investment
IFDI	Inward foreign direct investment
OFDI	Outward foreign direct investment
EQI	The comprehensive environmental quality index
EPI	The environmentally cleaner production index
ETI	The environmental end treatment index
SYS-GMM	A system-generalised method-of-moments
FE	Fixed effects model
CPC	The Communist Party of China
ASEAN	Association of Southeast Asian Nations
G	The overall Gini coefficient
G_w_	The intra-regional differences
G_nb_	The inter-regional differences
G_t_	The hypervariable densities
E-C	East–central
E-W	East–west
C-W	Central–west
Obs	Observed value
Std. Dev.	Standard deviation
CSY	China Statistical Yearbook
PSY	Provincial Statistical Yearbook
Wind	Wind database
R&D	Research and development
lnEQI	Ln (provincial comprehensive environmental quality index)
lnEPI	Ln (provincial environmentally cleaner production index)
lnETI	Ln (provincial environmental end treatment index)
lnIFDI	Ln (provincial IFDI flow)
lnOFDI	Ln (provincial OFDI flow)
ln(IFDI×OFDI)	Ln (provincial IFDI flow × provincial OFDI flow)
lnED	Ln (provinces’ GDP/population of each province)
IND	Provinces’ tertiary sector value added/provinces’ GDP
ES	Proportion of coal in energy consumption of each province
ER	Provinces’ pollution treatment cost/provinces’ added value of secondary industry
RD	Research and development investment: provinces’ R&D investment/provinces’ GDP
lnPOP	Ln (population density: population of each province/total provinces’ area)
